# A standardised method of marking male mosquitoes with fluorescent dust

**DOI:** 10.1186/s13071-020-04066-6

**Published:** 2020-04-15

**Authors:** Nicole J. Culbert, Maria Kaiser, Nelius Venter, Marc J. B. Vreysen, Jeremie R. L. Gilles, Jérémy Bouyer

**Affiliations:** 1 Insect Pest Control Laboratory, Joint FAO/IAEA Programme of Nuclear Techniques in Food and Agriculture, Seibersdorf, Vienna, Austria; 2 Institute of Integrative Biology & The Centre for Genomic Research, University of Liverpool, Liverpool, Merseyside UK; 3 Vector Control Reference Unit, National Institute for Communicable Diseases of the National Health Laboratory Service, Sandringham, Johannesburg, South Africa; 4 CIRAD, UMR ASTRE CIRAD-INRA, Animals, Health, Territories, Risks and Ecosystems, Campus International de Baillarguet, 34398 Montpellier, France

**Keywords:** Sterile insect technique, Dust, Diptera, Mark-release-recapture

## Abstract

**Background:**

Prior to a major release campaign of sterile insects, including the sterile insect technique, male mosquitoes must be marked and released (small scale) to determine key parameters including wild population abundance, dispersal and survival. Marking insects has been routinely carried out for over 100 years; however, there is no gold standard regarding the marking of specific disease-transmitting mosquitoes including *Anopheles arabiensis*, *Aedes aegypti* and *Aedes albopictus*. The research presented offers a novel dusting technique and optimal dust colour and quantities, suitable for small-scale releases, such as mark-release-recapture studies.

**Methods:**

We sought to establish a suitable dust colour and quantity for batches of 100 male *An. arabiensis*, that was visible both by eye and under UV light, long-lasting and did not negatively impact longevity. A set of lower dust weights were selected to conduct longevity experiments with both *Ae. aegypti* and *Ae. albopictus* to underpin the optimal dust weight. A further study assessed the potential of marked male *An. arabiensis* to transfer their mark to undusted males and females.

**Results:**

The longevity of male *An. arabiensis* marked with various dust colours was not significantly reduced when compared to unmarked controls. Furthermore, the chosen dust quantity (5 mg) did not negatively impact longevity (*P* = 0.717) and provided a long-lasting mark. Dust transfer was found to occur from marked *An. arabiensis* males to unmarked males and females when left in close proximity. However, this was only noticeable when examining individuals under a stereomicroscope and thus deemed negligible. Overall, male *Ae. aegypti* and *Ae. albopictus* displayed a greater sensitivity to dusting. Only the lowest dust weight (0.5 mg) did not significantly reduce longevity (*P* = 0.888) in *Ae. aegypti*, whilst the lowest two dust weights (0.5 and 0.75 mg) had no significant impact on longevity (*P* = 0.951 and 0.166, respectively) in *Ae. albopictus*.

**Conclusion:**

We have devised a fast, inexpensive and simple marking method and provided recommended dust quantities for several major species of disease-causing mosquitoes. The novel technique provides an evenly distributed, long-lasting mark which is non-detrimental. Our results will be useful for future MRR studies, prior to a major release campaign.
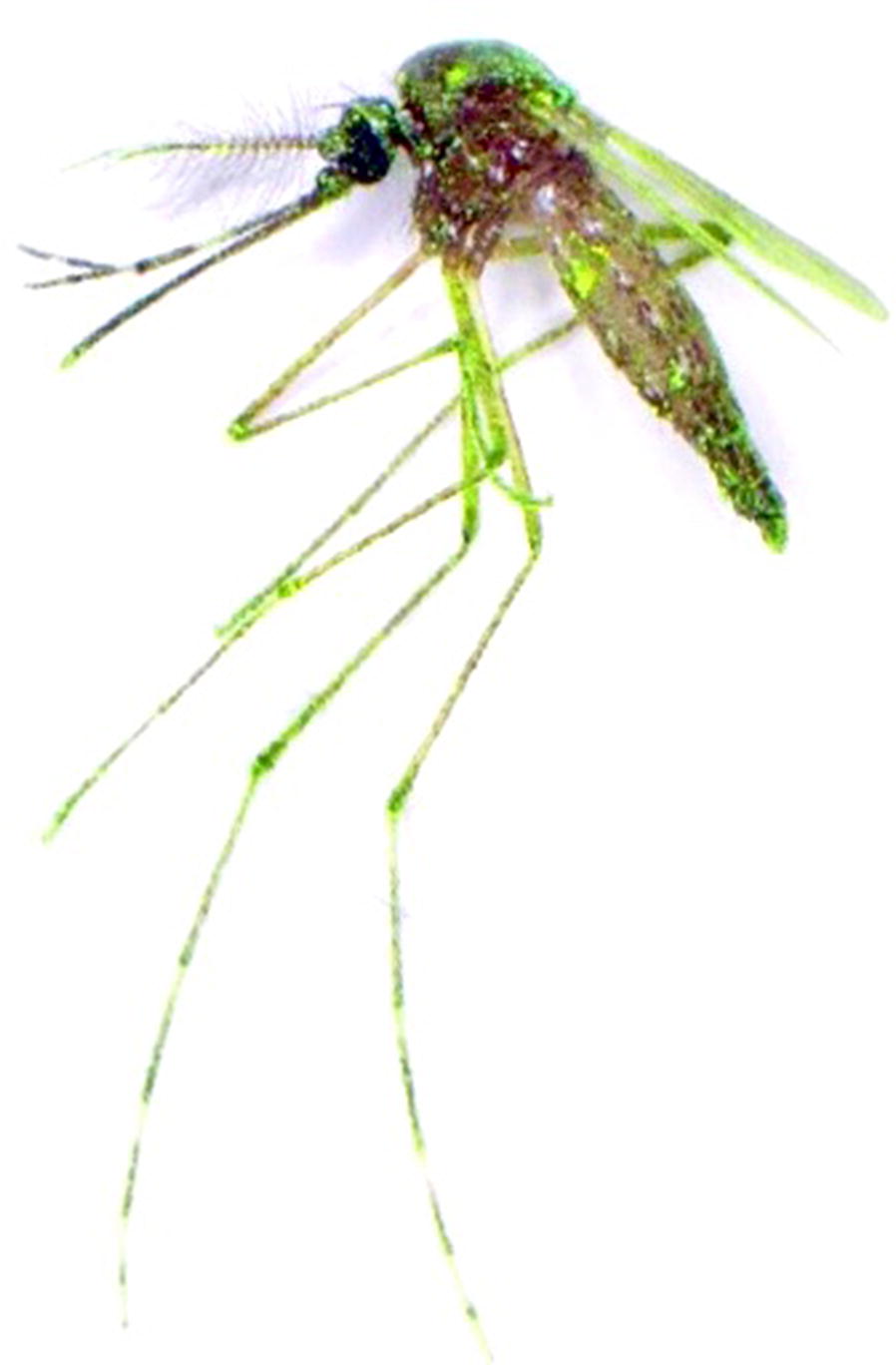

## Background

Marking insects for scientific studies has been ongoing for almost 100 years [1, 2]. Mark-release-recapture (MRR) studies are extremely useful and allow calculations of dispersal and mortality rates as well as providing estimates of population size. Historically, MRR experiments have prodigiously focused on assessing female survival and dispersal, due to their significant role in disease transmission, as highlighted in a recent review [3]. A rekindled interest in male mosquito genetic control programmes, such as the sterile insect technique (SIT) [4], has seen the focus shift towards male ecology and highlighted the need for more male MRR studies, in particular for estimating the competitiveness of irradiated sterile males in the field, which is still missing in the literature for mosquitoes [5].

Fluorescent paints [6], dyes [7, 8] and dusts [9, 10] in an array of colours are commonly used, in addition to methods involving the use of radio isotopes [11], trace elements [12], protein immunomarking [13] and genetic or transgenic techniques, including mutations leading to a distinguishable phenotypic difference or transfection of a symbiont such as *Wolbachia* [14–16]. Currently, there is no universal marking method applicable to all insect species. The suitability of the marking method will depend on several criteria such as the species and number of insects required to be marked, the environment the insect will encounter, the nature of the experiment, ease of application, and ultimately the cost of the method. The chosen method of marking can exert different effects between different species.

A critical component of the sterile insect technique (SIT) package is being able to monitor sterile males post-release and to distinguish them from wild males when collected in traps. Marking sterile insects prior to release is necessary to assess the efficiency of an SIT programme through a continuous assessment of the sterile to wild male ratio and is commonly achieved using MRR experiments. It is important to use a method of marking which is long-lasting, fast and easy to apply, in addition to being of low-cost, as within an operational level of an SIT programme, millions of sterile males may have to be marked at any one time. Moreover, the method of marking should have little or no effect upon the quality of the insect, with regard to competitiveness, flight ability and longevity.

Dusts or powders have been used to mark insects for more than 75 years [17] and are perhaps the most frequently used material [18]. The largest SIT programmes in the world involve rearing and releasing hundreds of millions of fruit flies on a weekly basis. For over five decades, fluorescent dust has been applied during the pupal stage, resulting in the emerging adults retaining their mark [19]. There are several types of fluorescent dusts that have been used in previous insect marking studies, from manufacturers such as Brilliant General Purpose [10], RADGLO [20] Brian Clegg [21] and DayGlo® [21]. DayGlo dusts are available in a broad spectrum of bright colours, allowing separate cohorts to be marked with different colours. Moreover, once applied, the dust is visible to the naked eye with enhanced detection under UV light. There are a variety of methods to apply dust to insects. Mosquitoes can be marked by placing them in a dusted plastic bag and gently shaking them. Previous studies that use a shaking procedure to mark delicate insects including mosquitoes have reported high mortality immediately after dusting in addition to coating them with too much dust [22]. Alternatively, a bulb duster can be used to puff dust on to the mosquitoes or a fan placed within a cage to create a dust storm [21]. Mosquitoes are usually immobilized prior to dusting as this increases the likelihood of a more uniform coverage of dust.

Despite the large volume of publications dealing with marking insects, there is no gold standard available when it comes to marking specific species of mosquitoes. The research presented within this paper aims to develop a fast, low-cost and low-effort marking procedure for the small-scale release of *Anopheles arabiensis*, *Aedes aegypti* and *Aedes albopictus*, three of the main vectors of mosquito-borne diseases, that does not negatively impact the quality of the insect. A preliminary trial was conducted with male *An. arabiensis* using blue, yellow and pink DayGlo® dust to determine which colour provided the best visibility, both under UV light and with the naked eye. After selecting a dust colour, various weights of dust were applied to male *An. arabiensis* to determine the lowest dust amount necessary to mark a known number. The impact of this dust amount on the survival of male *An. arabiensis* mosquitoes and its persistence over time was investigated. It is vital that there is minimal or no transfer of the mark between released sterile males and the wild population in order to obtain accurate data during trapping. Thus, a further study investigating the transfer of dust between marked and unmarked control male and female *An. arabiensis* was conducted. Two further studies were undertaken to investigate a range of dust weights and their impact upon the survival of male *Ae. aegypti* and *Ae. albopictus* mosquitoes in addition to the persistence of the mark over time.

## Methods

### Source of mosquito colonies and mass-rearing procedures

Experiments were carried out with mosquitoes from three established colonies. *Anopheles arabiensis* (Dongola strain), were sourced from field collections in the Northern State of Sudan and transferred to the Food and Agricultural Organisation/International Atomic Energy Agency (FAO/IAEA) Insect Pest Control Laboratory (IPCL) in Seibersdorf, Austria, by the Tropical Medicine Research Institute in Khartoum in 2005. *Aedes albopictus* (Rimini strain) were sourced in Rimini, Italy and transferred to the IPCL by the Centro Agricoltura Ambiente “G. Nicoli” in Crevalcore, Italy, in 2010. *Aedes aegypti* (Brazil strain) were sourced in Juazeiro, Brazil and transferred to the IPCL by Moscamed, Brazil, in 2012. All strains have been subsequently maintained at the IPCL under controlled temperature, relative humidity (RH) and light regimes (27 ± 1 °C, 70 ± 10% RH, 12:12 h light:dark (L:D) photoperiod with 1 h periods of simulated dawn and dusk). Eggs used for these experiments were generated following the *An. arabiensis* and *Aedes* rearing guidelines developed at the IPCL [23, 24]. *Anopheles arabiensis* larvae were mass-reared in plastic trays (100 × 60 × 3 cm) containing 4 litres of deionized water. Four thousand eggs were added per tray within a plastic ring floating on the water surface. Larvae were fed daily with 1% (wt/vol) IAEA diet developed and described in [25]. *Aedes* larvae were mass-reared in the same way as *An. arabiensis* larvae, but with 5 litres of water per tray and at a larval density of 18,000 first-instar (L_1_) and provided with 7.5% IAEA diet as detailed in [26].

### Pupae collection

*Anopheles arabiensis* pupae were manually separated from larvae using a cold-water vortex technique as described in [27] and males separated from females by observing the terminalia under a stereomicroscope [28]. *Aedes* pupae were sexed mechanically using a Fay-Morlan glass plate separator [29] as redesigned by Focks (John W. Hock Co., Gainesville, FL [30]). Male pupae were left to emerge inside 30 × 30 × 30 cm cages (BugDorm, Taipei, Taiwan) and provided with either a 5% (*An. arabiensis*) or 10% (*Aedes*) sucrose solution.

### Dust colour, optimized dust quantity and marking technique

The initial dust amounts tested with *An. arabiensis* were 1000 and 500 mg of dust per 100 male mosquitoes, based on the amounts used by Kluiters et al. [10] to mark *Culicoides* midges but these severely impacted immediate post-dusting survival. Therefore, a subsequent series of dust weights were investigated per 100 males and mortality assessed after 24 h, i.e. 100, 75, 50, 15, 10, 7.5, 6.3 and 5 mg of dust, with the lowest dust amount (5 mg) chosen as the optimal weight for all subsequent experiments. This amount was chosen as it provided an even coating of dust that was visible with both the naked eye and under UV light. A lower series of dust weights were chosen for determining the optimal amount to use for *Ae. aegypti* and *Ae. albopictus* males, (1.5, 1, 0.75 and 0.5 mg per 100 males), as it was discovered during the first marking session that 5 mg, despite marking adequately, left a surplus of dust behind. It was postulated that this may be due to their smaller body size as was noted when comparing the weight and volume occupied by 1000 males of all three aforementioned species in earlier laboratory tests, with both batches of male *Aedes* species weighing less than that of *An. arabiensis* (NJC, personal observation). Both *Aedes* and *An. arabiensis* males were marked at 48 hours-old.

Initially, three colours of fluorescent pigment were investigated (A-11, Aurora pink; A-17-N Saturn yellow; and A-19, Horizon blue), all from the DayGlo® (DayGlo Color Corp, Cleveland, USA) series as it is a brand routinely used to mark various other insects within the IPCL laboratory. Nine plastic urine cups (× 100 ml) were zeroed on an analytical balance and 5 mg of dust added to a cup, with 3 replicates per colour. After the addition of a plastic lid, the cups were shaken vigorously to coat the interior evenly. Twelve batches of 100 male mosquitoes were immobilized at 4 °C. All batches were transferred to the pre-dusted cups *via* a mouth aspirator. The cups were then gently rotated for 30 s, equating to 25 full rotations, to ensure all mosquitoes were evenly coated. The remaining 3 batches were rolled inside an undusted cup and served as controls. All males were returned to their original Bugdorms, maintained within the laboratory, whilst still inside their cups. The lid was removed, the cup placed on its side and the mosquitoes given sufficient time to recover before the cup was removed.

### Marking and adult male longevity

The impact of marking on male *An. arabiensis* longevity was assessed by comparing marked experimental with unmarked control males. Six batches of 100 male pupae were sexed under a stereomicroscope and allowed to emerge in Bugdorm cages with access to a sugar solution. All batches were immobilized and dusted as previously described. Three batches were marked with 5 mg of dust with the remaining 3 left undusted and serving as controls. Survival was monitored by removing dead individuals daily until all cages were empty on day 47. *Aedes* males were marked as described above for *An. arabiensis* but with 1.5, 1, 0.75 or 0.5 mg per 100 adults. Survival was monitored for 28 days post-dusting.

### Dust persistence over time

To investigate the persistence of dust over time in *An. arabiensis*, 3 groups of 500 males were immobilized and marked as previously described and released into large field cages (1.8 m^2^) with a sugar solution provided. Two black plastic cups (500 ml) were placed inside each cage. One in a horizontal and one in a vertical position. Every second day, a lid was placed over each cup prior to removal to determine how many mosquitoes were inside. A photograph of each cup was taken to assess the persistence of the mark over time. All mosquitoes were then released into a fourth cage, to prevent resampling of the population, until few or no mosquitoes were collected in the black cups for several subsequent days.

### Dust transfer between marked and unmarked adults

Marked males were caged with unmarked males and females to determine whether they are capable of transferring dust. Pupae were sexed under a stereomicroscope into sets of 100 to populate 9 large Bugdorm cages (30 × 30 × 30 cm) containing 100 of each sex. A further 9 sets of 100 males were sexed and allowed to emerge in small Bugdorm cages (15 × 15 × 15 cm). All cages contained a sugar solution. The 9 small cages of males were immobilized and dusted with 5 mg of dust as previously described. Each set of dusted males was then transferred to a large Bugdorm cage containing 100 undusted males and females. After 1 day, all marked males were then carefully aspirated out of 3 randomly selected cages before the cages were placed in a − 20 °C freezer to kill all remaining mosquitoes. Males and females were then screened under a stereomicroscope to check for the presence of dust particles. On day 3 post-dusting, this step was repeated with an additional 3 cages and again on day 7 with the remaining 3 cages.

### Statistical analysis

Binomial linear mixed effect models were used to analyse the impact of the various dust treatments on survival (response variable). The dust treatments were used as fixed effects whilst the replicates were set as random effects. The significance of fixed effects was tested using the likelihood ratio test [31, 32]. Fixed-effects coefficients of all models and their corresponding *P*-values are reported in Additional file 1: Tables S1–S5.

## Results

### Dust colour and optimised dust quantity

The longevity of experimental *An. arabiensis* males did not significantly differ from undusted control males when dusted with blue (*P* = 0.217), yellow (*P* = 0.804) or pink (*P* = 0.335) fluorescent dust (Additional file 1: Table S1). A-11 Aurora pink was chosen as the dust colour for all further experiments as it was the most distinguishable colour under a UV microscope and with the naked eye. A dose of 5 mg for 100 males was selected as the optimal dust quantity for *An. arabiensis* after testing a range of dust (blue, yellow or pink) quantities in a preliminary study (Table 1).

**Table 1:** Dust coverage and mortality 24 hours after dusting 100 male *Anopheles arabiensis*

Dust amount (mg)	Mortality	Dust coverage
1000	High	Very heavy
500	High	Very heavy
100	High	Very heavy
75	High	Very heavy
50	High	Very heavy
15	Medium	Heavy
10	Medium	Heavy
7.5	Medium	Medium
6.3	Medium	Medium
5	Low	Good

*Note*: High > 50% mortality; medium, 10–50% mortality; low, < 10% mortality

### Longevity of dusted adult males and dust persistence over time

**Fig. 1:**
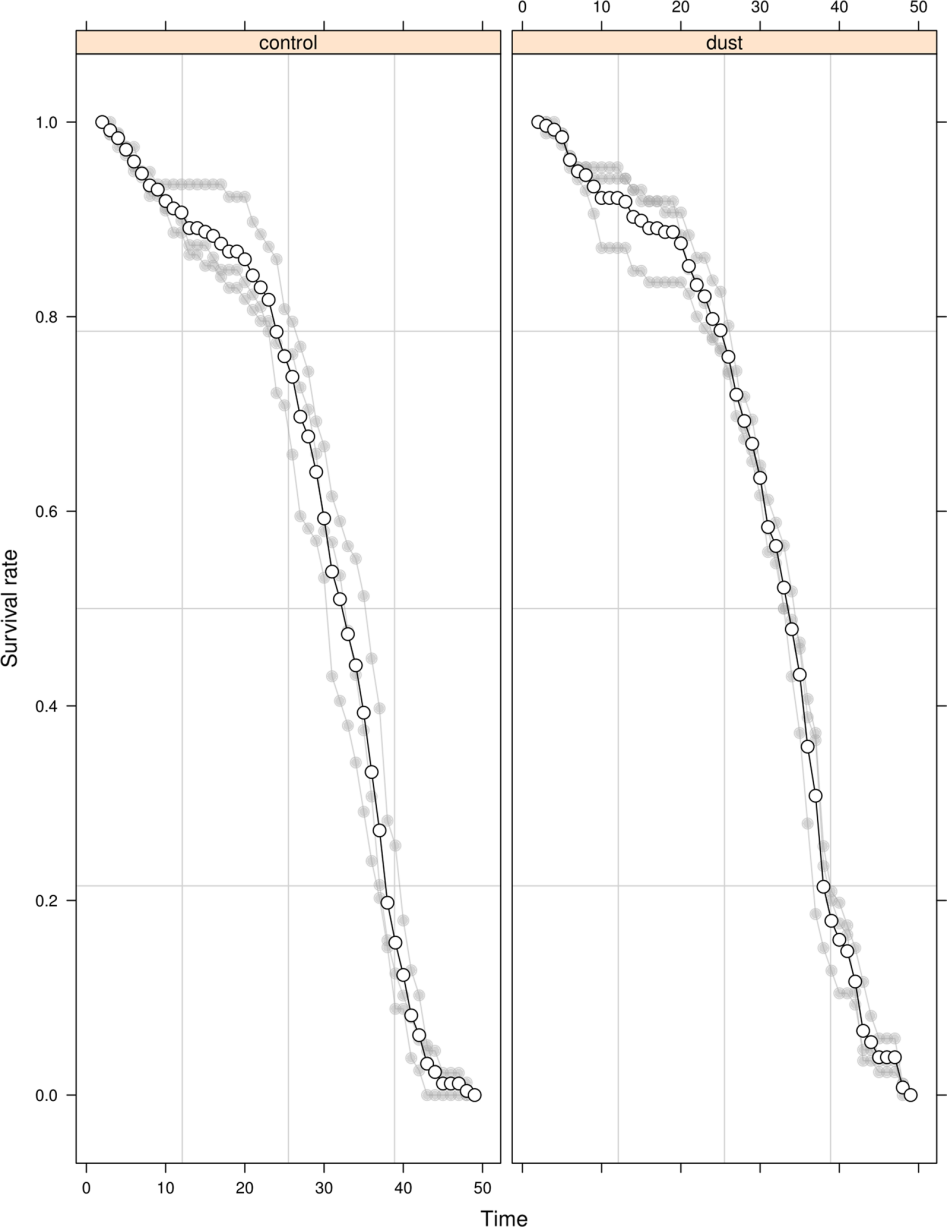
The longevity of pink dusted male *Anopheles arabiensis* and undusted controls over time. Survival was assessed for 5 mg of pink fluorescent dust for 100 males. Individual values of the replicates are indicated in light grey and mean values as a solid line

**Fig. 2:**
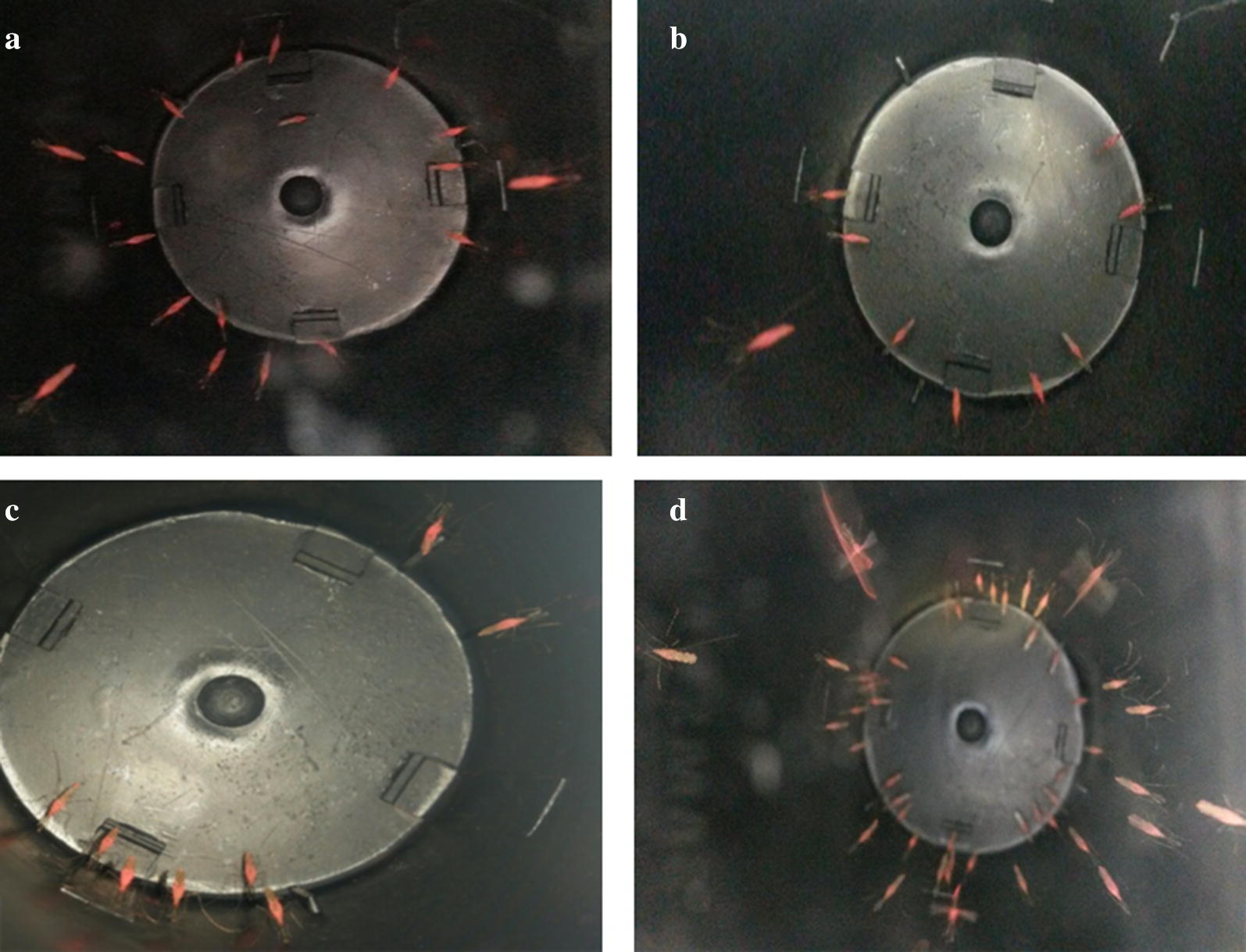
Photographs depicting presence of 5 mg of pink dust over 28 days. **a** 2 days. **b** 8 days. **c** 12 days. **d** 28 days

The longevity of dusted *An. arabiensis* males (5 mg/100 males) was not significantly different from that of undusted controls (*P* = 0.717, Fig. 1, Additional file 1: Table S2), with both control and dusted males surviving up to day 48. Photographs taken every second day showed that the selected dust amount (5 mg/100 adults) remained visible on marked males for upwards of one month (Fig. 2).

**Fig. 3:**
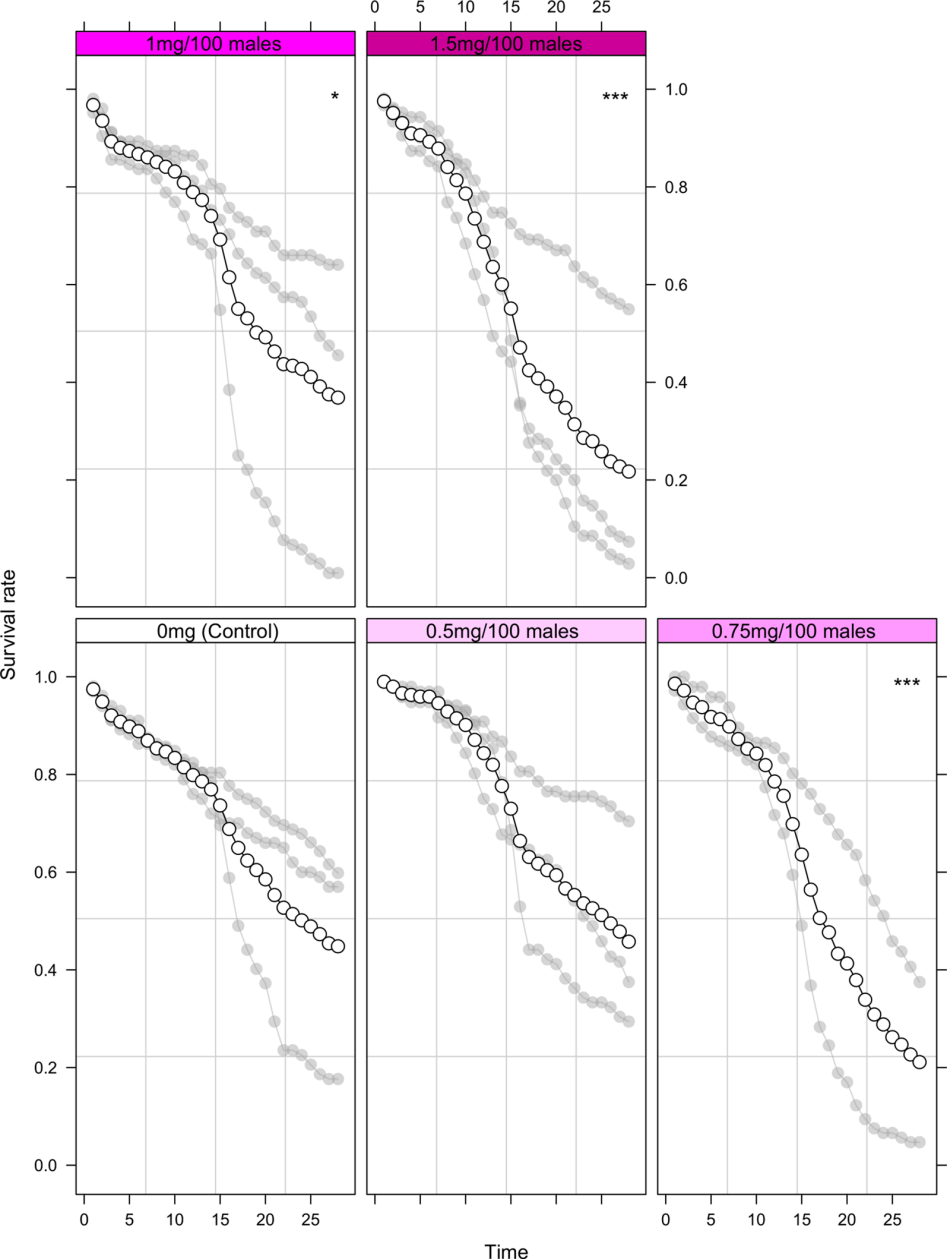
Longevity of male *Aedes aegypti* marked with various pink dust quantities over 28 days. Significant differences between experimental males (0.5, 0.75, 1 and 1.5 mg) and the control group (no dust) are indicated (*P < 0.05, **P < 0.01, ***P < 0.001). Individual values of the replicates are indicated in light grey and mean values as a solid line

**Fig. 4:**
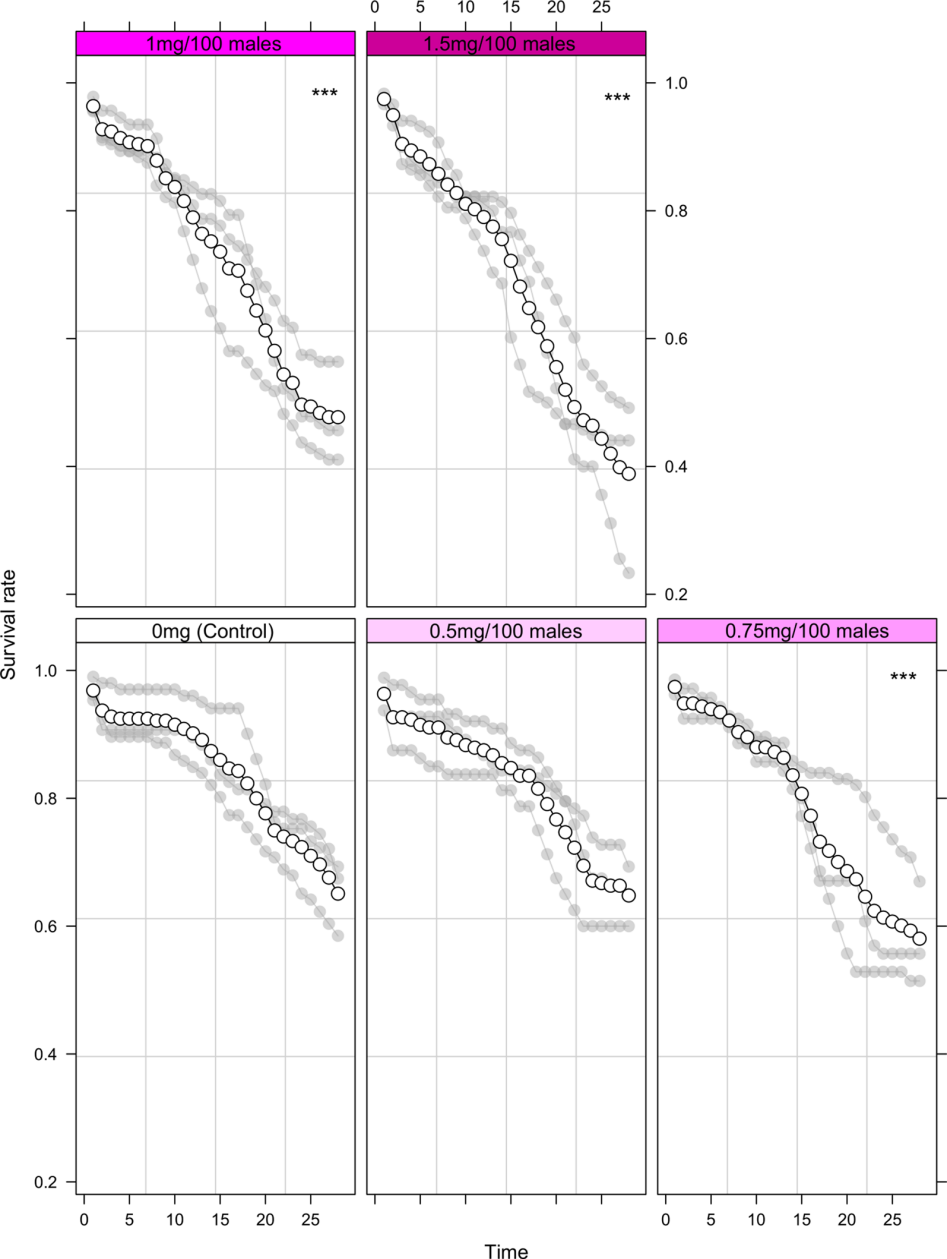
Longevity of male *Aedes albopictus* marked with various pink dust quantities over 28 days. Significant differences between experimental males (0.5, 0.75, 1 and 1.5 mg) and the control group (no dust) are indicated (*P < 0.05, **P < 0.01, ***P < 0.001). Individual values of the replicates are indicated in light grey and mean values as a solid line

Results indicated that only the lowest dust amount (0.5 mg) did not significantly decrease longevity in male *Ae. aegypti* when compared to undusted controls (*P* = 0.888, Fig. 3, Additional file 1: Table S3). Interestingly, *Ae. albopictus* appeared less sensitive to dusting with the lowest two dust amounts (0.5 and 0.75 mg) having no significant impact on longevity in comparison to undusted controls (*P* = 0.951 and 0.166 respectively, Fig. 4, Additional file 1: Table S4).

**Fig. 5:**
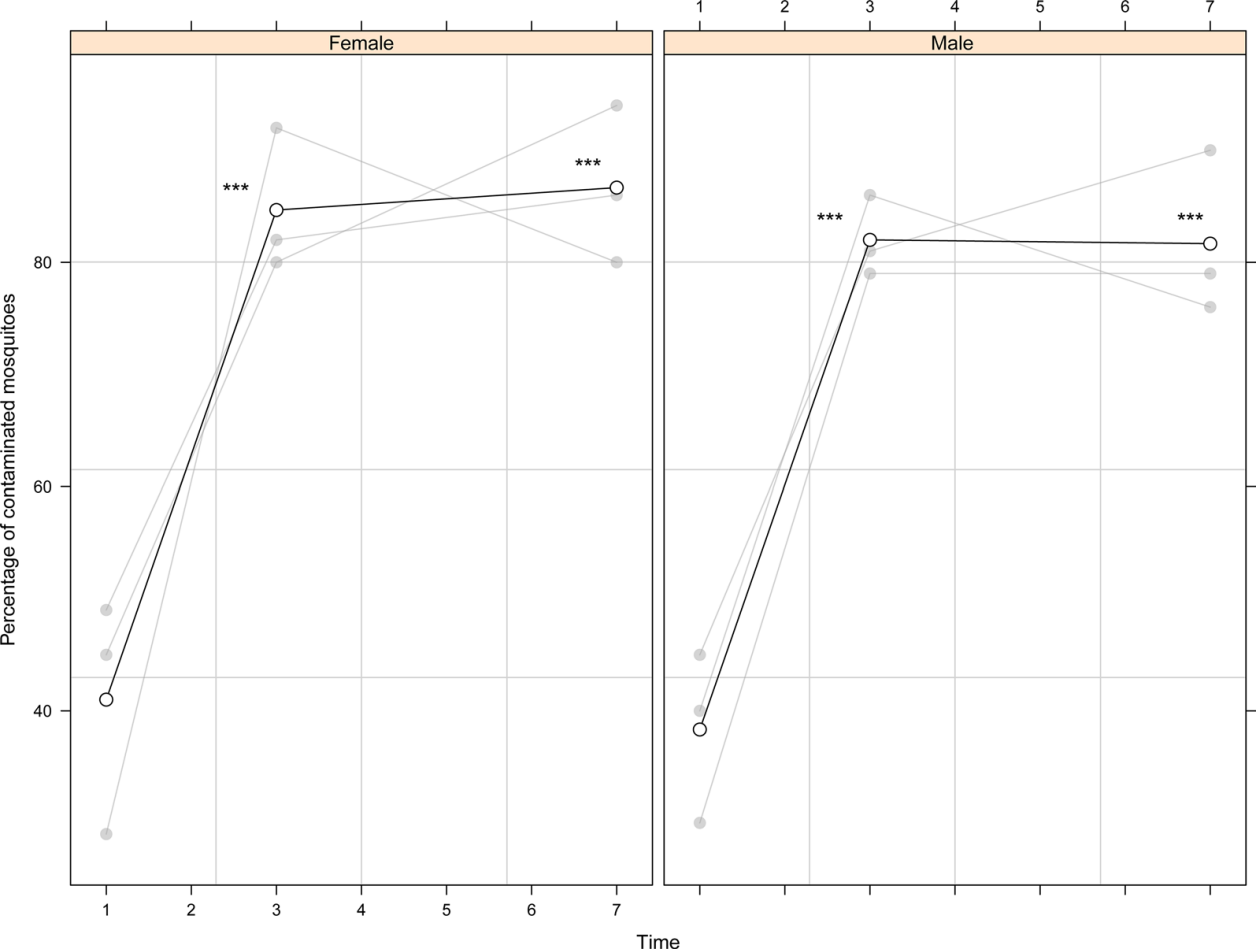
Dust transfer from marked male *Anopheles arabiensis* to unmarked males and females. 100 marked males (5 mg/100) were caged with 100 unmarked males and females. On days 1, 3 and 7 dusted males were removed from 3 cages and the remaining males and females screened for dust. Significant differences between the percentage of male and female mosquitoes contaminated with dust are indicated (*P < 0.05, **P < 0.01, ***P < 0.001). Individual values of the replicates are indicated in light grey and mean values as a solid line

Visual inspection under a stereomicroscope was required to assess whether dust had been transferred to unmarked males and females as it was not noticeable either by the naked eye or under a UV light. There was no significant effect of sex on whether dust was transferred (*P* = 0.091) but there were significantly more males and females displaying dust transfer on days 3 and 7 (*P* < 0.001) in comparison to day 1 (Fig. 5, Additional file 1: Table S5).

## Discussion

The experimental work presented in this paper has allowed us to determine some key parameters relevant to developing a standardized small-scale method of marking *An. arabiensis*, *Ae. aegypti* and *Ae. albopictus* male mosquitoes prior to a release campaign such as that of a mark-release-recapture (MRR) study. We selected a dust colour, optimal amounts for all three species and verified that it did not impact mosquito longevity, nor was the mark easily transferred.

In addition to increasing immediate mortality, applying too much dust to an insect poses the problem that it cannot groom the excess off, thus decreasing mobility and interfering with their sensory organs. Thus, our first challenge was to determine the lowest possible amount of dust necessary to achieve a mark both visible to the naked eye and under UV light. Following this, we tested various colours with this dust amount to establish the most appropriate one. Different studies aimed at assessing the effect of different fluorescent dust colours on mosquito longevity have reported mixed results. Some studies indicate no difference in longevity between various dust colours [33] whereas others conclude that different colours exert varying effects upon mosquito longevity. One study noted blue as being particularly detrimental to mosquito longevity, even when various manufacturers of blue dust were tested. The authors also stated that blue dust is less visible after application in comparison to other colours [21]. No significant difference in longevity was noted after comparing the longevity between marked male *An. arabiensis* with different colours and unmarked controls. However, the blue and yellow mark were much less visible by eye and under UV light thus pink was chosen as the marking colour to be used for our marking protocol. Furthermore, previous studies have also shown pink or red dusts to exert less of an impact upon longevity when compared to other colours [21].

After selecting our dust amount (5 mg/male) and colour (pink) for *An. arabiensis*, we verified that it did not negatively impact the longevity of marked males, which is consistent with what was stated by Dickens & Brant [21]. It is important to ensure that marked insects retain their mark for an appropriate amount of time. Once released, it will be necessary to identify sterile males and distinguish them from wild counterparts. We were able to successfully verify that our dust amount was sufficient to retain the visibility of the mark for upwards of one month, both with the naked eye and a UV light. We adopted to extrapolate our technique to *Ae. aegypti* and *Ae. albopictus*; however, due to their smaller body size, we concluded that a lower dust quantity would be required. The results of our study confirmed this initial postulation to be correct, with only the lowest dust weight of 0.5 mg found not to significantly decrease survival rate in *Ae. aegypti.* Our marking method proved successful when it was up scaled and used to mass-mark male *Ae. aegypti* prior to aerial release as part of a mark-release-recapture (MRR) study undertaken in Brazil in 2018 (unpublished data). The amount of dust used to coat 100 males (0.5 mg) was increased to 12 mg to mark batches of 2400 males in 1 litre buckets and followed the same experimental guidelines used for small-scale marking experiments in the laboratory. In total, the technique was used to mark over 250,000 sterile males. Dickens & Brandt [21] also found a significant decrease in survival in marked male *Ae. aegypti* in comparison to unmarked controls, although this result may not be surprising when considering they used 0.3 g of dust per 30 adult males [21]. Male *Ae. albopictus* were less sensitive to dusting however with mortality rates for 0.5 and 0.75 mg per 100 similar to undusted controls. Marini et al. [34] also reported no significant impact upon survival when dusting male *Ae. albopictus* prior to release as part of a MRR study in Italy, although dust weight was not reported.

It is important that the mark is not easily transferable to the wild population of either males or females. The literature tends to suggest that fluorescent dust does not transfer between marked and unmarked mosquitoes when held together thus we conducted our own investigation to clarify or disprove this finding. A previous study conducted by Kluiters et al. [10] found that dust was not transferred when 30 marked and 30 unmarked *Culicoides* were confined for 24 hours within a trapping beaker. Fryer & Meek [35] found that only 3% of unmarked *Psorophora columbiae* mosquitoes (9 out of 300) became marked during a 24-hour period of being caged with marked adults. This result further confirmed reports from an earlier study which reported no dust transfer during the mating of *Drosophila pseudoobscura* or following heavy crowding of marked and unmarked individuals. However, marked insects were given time to groom themselves following dusting [36].

In stark contrast to the literature, our results indicated that marked males are indeed capable of transferring the mark to both males and females and in addition to this, the percentage of non-marked individuals that became marked increased over time. However, the dust transferred was not visible with the naked eye or under UV light. It was detectable only *via* examination with a stereomicroscope. We aimed to clarify the dust particle number transferred, which proved impossible as in most cases, the transferred dust was in a clump. Thus, even though we found a high percentage of unmarked males and females showing evidence of dust transfer, it is unlikely to be relevant for sterile males marking wild males or females post-release. However, it is an encouraging finding for techniques such as boosted SIT, which relies on the close contact of sterile males (coated with pyriproxyfen powder or densovirus) and wild females during mating and the transfer of powder [37, 38]. A marked male is very easily distinguishable from a male or female which has evidence of dust transfer. It is likely that when recollecting sterile males, UV light will be used to detect their presence following recollections from traps and subsequent examinations within a laboratory setting. Dust transfer was not detectable in our study, unless using a stereomicroscope.

The short duration (24 hours) of holding marked and unmarked insects together may go some way to explain the lack of dust transfer in the above two studies. Or the close confinement of our marked and unmarked individuals in 30 × 30 × 30 cm bugdorm cages may explain why we saw such a high level of dust transfer. Alternatively, it may be the behaviour of this mosquito species itself that caused such a degree of dust transfer, as male *An. arabiensis* form swarms when mating [39]. It would be beneficial to repeat this experiment in large field cages to determine if indeed a limited spatial environment was responsible or by allowing the marked males sufficient time for grooming following dusting. However, it is likely that in a mass-rearing facility, if immobilizing sterile males for marking, they will be packed into release canisters immediately afterwards, thus not allowing a window of time for grooming. If mosquitoes are self-marked or marked whilst active, it would then be possible to allow them time to groom prior to immobilizing them for packing.

The results of our study demonstrated that immobilizing male mosquitoes prior to dusting does not significantly impact their survival. However, depending on the type and scale of marking experiment being undertaken, immobilizing mosquitoes prior to marking may not always be feasible. Marking active male *Ae. albopictus* mosquitoes prior to a series of MRR experiments in Mauritius, has been shown to be effective [4]. Thus, our technique should be seen as another ‘tool’ in the SIT toolbox as opposed to the only or best available method.

There is much conflicting information in the literature regarding the use of fluorescent dust to mark mosquitoes. There does seem to be a general consensus that the dust colour and manufacturer can impact mosquito longevity negatively, in addition to the technique used to apply the dust. The main aim of our study was to provide a standardized guide to dust-marking several of the key disease-causing vectors of mosquito to deployed in small-scale releases of sterile male mosquitoes such as MRR studies. Our results highlight how determining the optimal dust quantity for one species, for example *An. arabiensis*, does not automatically mean that it can be inferred for another species (*Ae. aegypti*).

## Conclusions

We have devised a fast, inexpensive and straightforward method of marking immobile male mosquitoes and provided recommended dust quantities for several major species of disease-causing mosquitoes. Our marking technique provides an evenly distributed mark which is long-lasting and non-detrimental. When attempting to deploy this technique in the future on a yet to be tested species of mosquito, it is recommended to perform longevity experiments with several dust quantities to ascertain the optimal concentration for the studies species.

## Supplementary information

**Additional file 1: Table S1.** Fixed-effects coefficients of a mixed-effect binomial model investigating the impact of dust colour on survival in *An. arabiensis.***Table S2.** Fixed-effects coefficients of a mixed-effect binomial model investigating the impact of pink dust (5 mg/100) on survival in *An. arabiensis.***Table S3.** Fixed-effects coefficients of a mixed-effect binomial model investigating the impact of dust quantity on survival in *Ae. aegypti.***Table S4.** Fixed-effects coefficients of a mixed-effect binomial model investigating the impact of dust quantity on survival in *Ae. albopictus.***Table S5.** Fixed-effects coefficients of a mixed-effect binomial model investigating the occurrence of dust transfer between pink dusted (5 mg/100) male *An. arabiensis* and undusted males and females after 1, 3 and 7 days.

## Data Availability

All data generated or analyzed during this study are included in this published article.

## Abbreviations

SIT: sterile insect technique
RH: relative humidity
IPCL: Insect Pest Control Laboratory
FAO: Food and Agricultural Organization
IAEA: International Atomic Energy Agency
LD: light:dark
MRR: mark-release-recapture
