# Supplementary material for: A standardised method of marking male mosquitoes with fluorescent dust
Source: Parasit Vectors. 2020 Apr 15;13:192. doi: 10.1186/s13071-020-04066-6 (PMC7158013; doi:10.1186/s13071-020-04066-6)
Supplement: Supplementary file 1 — Additional file 1: Table S1. Fixed-effects coefficients of a mixed-effect binomial model investigating the impact of dust colour on survival in An. arabiensis.Table S2. Fixed-effects coefficients of a mixed-effect binomial model investigating the impact of pink dust (5 mg/100) on survival in An. arabiensis.Table S3. Fixed-effects coefficients of a mixed-effect binomial model investigating the impact of dust quantity on survival in Ae. aegypti.Table S4. Fixed-effects coefficients of a mixed-effect binomial model investigating the impact of dust quantity on survival in Ae. albopictus.Table S5. Fixed-effects coefficients of a mixed-effect binomial model investigating the occurrence of dust transfer between pink dusted (5 mg/100) male An. arabiensis and undusted males and females after 1, 3 and 7 days. [file 13071_2020_4066_MOESM1_ESM.docx]

**Additional file 1: Table S1.** Fixed-effects coefficients of a mixed-effect binomial model of the impact of dust colour on survival in *Anopheles arabiensis*

| Fixed effects | Value | Std. Error | z-value | p-value |
| --- | --- | --- | --- | --- |
| Intercept | 0.8242 | 0.1385 | 5.951 | 2.67e-09 |
| blue | -0.3336 | 0.2700 | -1.235 | 0.217 |
| yellow | -0.0704 | 0.2837 | -0.248 | 0.804 |
| pink | -0.2646 | 0.2744 | -0.964 | 0.335 |

**Additional file 1: Table S2.** Fixed-effects coefficients of a mixed-effect binomial model of the impact of pink dust (5mg/100) on survival in *Anopheles arabiensis*

| Fixed effects | Value | Std. Error | z-value | p-value |
| --- | --- | --- | --- | --- |
| Intercept | 0.7425 | 0.1367 | 5.433 | 5.56e-08 |
| 5 mg | 0.0698 | 0.1923 | 0.363 | 0.717 |

**Additional file 1: Table S3.** Fixed-effects coefficients of a mixed-effect binomial model of the impact of dust quantity on survival in *Aedes aegypti*

| Fixed effects | Value | Std. Error | z-value | p-value |
| --- | --- | --- | --- | --- |
| Intercept | -0.19665 | 0.17341 | -1.134 | 0.2568 |
| 0 5 mg | 0.02297 | 0.16374 | 0.140 | 0.8884 |
| 0.75 mg | -1.10257 | 0.21322 | -5.171 | 2.33e-07 |
| 1 mg | -0.35453 | 0.16480 | -2.151 | 0.0315 |
| 1.5 mg | -1.17341 | 0.18497 | -6.344 | 2.24e-10 |

**Additional file 1: Table S4.** Fixed-effects coefficients of a mixed-effect binomial model of the impact of dust quantity on survival in *Aedes albopictus*

| Fixed effects | Value | Std. Error | z-value | p-value |
| --- | --- | --- | --- | --- |
| Intercept | 0.60744 | 0.13547 | 4.484 | 7.33e-06 |
| 0.5 mg | 0.01104 | 0.18052 | 0.061 | 0.951 |
| 0.75 mg | -0.24543 | 0.17699 | -1.387 | 0.166 |
| 1 mg | -0.71689 | 0.16886 | -4.246 | 2.18e-05 |
| 1.5 mg | -1.01447 | 0.16680 | -6.082 | 1.19e-09 |

**Additional file 1: Table S5.** Fixed-effects coefficients of a mixed-effect binomial model of the occurrence of dust transfer between pink dusted (5 mg/100) male *Anopheles arabiensis* and undusted males and females after 1, 3 and 7 days

| Fixed effects | Value | Std. Error | z-value | p-value |
| --- | --- | --- | --- | --- |
| Intercept | -0.25087 | 0.13310 | -1.885 | 0.0595 |
| Sex | -0.18420 | 0.10912 | -1.688 | 0.0914 |
| Day | 0.35680 | 0.02603 | 13.707 | <2e-16 |
